# Changes of serum midkine as a dynamic prognostic factor to monitor disease status in papillary thyroid cancer

**DOI:** 10.1097/MD.0000000000012242

**Published:** 2018-09-07

**Authors:** Ning Li, Chunmei Zhang, Zhaowei Meng, Ke Xu, Xianghui He, Yang Yu, Qiang Jia, Xue Li, Xiangxiang Liu, Xiaoran Wang

**Affiliations:** a Department of Nuclear Medicine; b Tianjin Key Laboratory of Lung Cancer Metastasis and Tumor Micro-environment, Tianjin Lung Cancer Institute; c Department of General Surgery, Tianjin Medical University General Hospital; d Department of Thyroid and Neck Tumor, National Clinical Research Center for Cancer, Key Laboratory of Cancer Prevention and Therapy, Tianjin Clinical Research Center for Cancer, Tianjin Medical University Cancer Institute and Hospital, Tianjin, P.R. China.

**Keywords:** ^131^I treatment, differentiated thyroid cancer (DTC), midkine (MK), papillary thyroid cancer (PTC), thyroglobulin (Tg)

## Abstract

This study aimed to investigate the value of dynamic changes of midkine (MK) to monitor post-surgical patients with papillary thyroid cancer (PTC) who were managed with ^131^I therapies.

MK concentration at initial ^131^I ablation therapy (MK1) as well as 10 to 12 months thereafter (MK2) was evaluated. And the dynamic changes of thyroglobulin (Tg) were compared (Tg1 and Tg2). Patients with MK influencing co-morbidities and with positive thyroglobulin antibodies were excluded. Concentrations of MK were measured by enzyme-linked immunosorbent assay.

There were 241 PTC patients (36 males, 205 females) enrolled, 55 cases had metastases (8 males, 47 females) during their follow-up. Cox regression showed if Tg2 decreased (compared with Tg1), but not to less than 1.0ng/mL under TSH stimulation, the risk of metastases was 12.554 times more than if it could decrease to the optimal level. If Tg2 increased, the risk is 19.461 times higher. As for MK, if MK2 level decreased (compared with MK1), but not to a normal level, the risk of metastases is 3.006. If MK2 level increased, it would be 5.030 likely to had metastases.

Our results indicated that MK could potentially be used as a disease monitoring biomarker for PTC, although inferior to Tg.

## 1 Introduction

Thyroid cancer is becoming a prevalent malignancy worldwide, not only in the USA,^[1]^ but also in China.^[2]^ Serum thyroglobulin (Tg) measurement is considered crucially important for the treatment and follow-up management of patients with differentiated thyroid cancer (DTC).^[3]^ However, Tg is not perfect under all circumstances. For instance, a technical interference from thyroglobulin antibodies (TgAb) can cause a falsely low or undetectable serum Tg, invalidating the value of Tg.^[4]^ The prevalence of TgAb in patients with DTC has been reported to vary between 8% to 36%, which is nearly 2-fold higher than in the general population.^[5]^ So, it is mandatory that TgAb is measured in all specimens sent for Tg testing.^[3]^ In contrast, the human antimouse antibodies (HAMA) or heterophile antibodies (HAB) interference usually results in a falsely high serum Tg level.^[6–7]^ This phenomenon is due to the innate attributes of the currently popular measuring methodology for Tg, the immunometric assay.^[4]^ Because HAMA or HAB can bind to animal antigens or antibodies employed in immunometric assay to form a bridge between the capture and detection antibody, whether in present or in absent of analyte.^[8]^ The prevalence of HAMA or HAB in patients with DTC can range from 0.4% to 10%.^[6–7,9]^ A false positive Tg may prompt unnecessary intervention on the disease.

Several attempts have been made to solve the above-mentioned problems, 1 of which is to employ some other novel serum markers. Midkine (MK) is a good example. As a pleiotropic growth factor, MK is prominently expressed during embryogenesis, which regulates cell growth, survival, migration, angiogenic, and anti-apoptotic activities.^[10–11]^ Nevertheless, during adulthood, MK is usually down-regulated to a very low level in healthy individuals. The advantage of MK is that it is a soluble cytokine, which is easily measurable in the blood circulation, making it a relatively convenient and non-invasive biomarker. Furthermore, a diagnostic kit with MK quantification is in a current stage of receiving regulatory clearance to enter the clinics.^[12]^ However, the disadvantage of MK is also obvious. In various pathological conditions, most notably in various cancers, strikingly enhanced MK over-expression has been noted.^[12]^ So, in other words, specificity of MK is low.

As far as thyroid cancer is concerned, 4 previous immunohistochemical studies ^[13–16]^ on the topic of papillary thyroid cancer (PTC) identified strong MK expression, which correlated with PTC clinicopathological characteristics as well as synchronous metastases. One serological investigation ^[17]^ indicated that MK could potentially be used to screen patients with thyroid nodules for DTC and to predict whether metastases exist before ^131^I ablative therapy. Kuzu et al ^[18]^ confirmed that both serum MK and nodular MK could predict tumorigenesis of thyroid nodules with highly malignant/suspicious thyroid cytopathology and sonographic features. In addition, Jee et al ^[19]^ studied thyroid nodule fine-needle aspiration samples and demonstrated that the MK/Tg ratio in PTC was greater than in benign thyroid nodules, providing adjunctive diagnostic or prognostic information to existing approaches. Our most recent study ^[20]^ found that MK could also be used as a surrogate biomarker for predicting DTC metastases in case of Tg invalidity due to TgAb interference. So, it would be interesting to know whether dynamic changes of MK could be a prognostic factor to predict disease status in DTC as well.

In this research, we aimed to evaluate the prognostic value of dynamic changes of MK to predict disease status in patients with PTC, who were treated with ^131^I during the studying period. Comparison between MK and Tg were also to be performed.

## 2 Patients and methods

### 2.1 Recruitment

During the recruitment period starting from January 2011, DTC patients admitted in Nuclear Medicine Department in our institution for the purpose of ^131^I treatment were asked to consider entering the MK clinical investigation. If they consented, DTC patients would be subjected to the measurements of serum MK along with Tg and TgAb concentrations consequently during every ^131^I treatment in approximately every 5 to 6-month follow-up intervals. For the purpose of the current investigation, a minimum of 2-year follow-up was required. So the ending of recruitment time point was January 2015. Detailed protocol was also described previously.^[17,20]^ MK database archive retrieval (from January 2011 till January 2015) was performed with the inclusion criterion of a confirmed post-surgical diagnosis of PTC in pathology. Exclusion criteria were

1) PTC patients with MK influencing co-morbidities like other oncologies, ischemic diseases, autoimmune diseases, kidney diseases, neural diseases, inflammation, hypertension, diabetes,
2) other post-surgical thyroid cancer pathology (such as anaplastic thyroid carcinoma, poorly PTC, follicular thyroid carcinoma),
3) prior ^131^I therapy TgAb was positive, and
4) complete follow-up data not available.

### 2.2 Ethics

The ethical and methodological aspects of this investigation were approved by the Institutional Review Board of Tianjin Medical University General Hospital. Written informed consents were provided by the participants to enter in this study. We confirm that all methods were performed in accordance with the relevant guidelines and regulations.

### 2.3 Management

Management protocols and procedures for the PTC patients were done generally according to the 2009 American Thyroid Association guideline.^[3]^ After a preparation of thyroid hormone withdrawal, each patient was given a therapeutic dose of ^131^I for every therapy. Blood tests were scheduled less than 2 days prior ^131^I therapy. Four to 5 days after ^131^I administration, whole body scan (as well as tomography imaging if necessary) was conducted by a dual-detector SPECT/CT machine with high-energy collimators. A Discovery VH SPECT/CT machine (General Electric Medical Systems, Milwaukee Wisconsin) was used for imaging before September 2014. Afterward, a new SPECT/CT machine, Discovery NM/CT 670 (General Electric Medical Systems), was installed and used for imaging. Diagnosis of disease status was made by our panel of nuclear medicine physicians in consensus, based on a comprehensive consideration of imaging, serological and other clinical materials, as reported previously.^[17,20,35]^ And we gave a therapeutic and follow-up recommendation for each patient. All patients were closely followed. Repeated ^131^I therapies were implemented with intervals of approximately 5 to 6 months to those with confirmed ^131^I-avid metastasis, or a diagnostic ^131^I scan to those without evidence of metastasis. After another ^131^I scan was performed, re-evaluation of disease status for each PTC patient was done again. Renewal of disease status was performed by our panel based on a series of tests and imaging results during every ^131^I treatment or diagnostic ^131^I scan. Final disease-free status was defined as stimulated Tg less than 1.0ng/mL under thyrotropin stimulation, negative TgAb, and no evidence of tumor on ^131^I scan or cervical ultrasound at the end of every patient's follow-up. Metastasis was defined as ^131^I-avid lesions on the ^131^I scans and elevated Tg levels.^[17,20,35]^ In most of the cases, metastatic lesions were also confirmed by cervical ultrasound, cervical CT, and lung CT.

### 2.4 Parameter measurement

A fully automated ADVIA Centaur analyzer (Siemens Healthcare Diagnostics) was used to measure free triiodothyronine (FT3), free thyroxine (FT4), and thyrotropin. A fully automated IMMULITE 2000 analyzer (Siemens Healthcare Diagnostics) was applied to assess Tg and TgAb. All these assays were based on a chemiluminescent reaction principle.

The calibration references for the above indices were: FT3, 3.50 to 6.50 pmol/L; FT4, 11.50 to 23.50 pmol/L; thyrotropin, 0.30 to 5.00 μIU/mL; Tg, 0 to 55.00 ng/mL (maximum measurable level 300.00 ng/mL); TgAb, 0 to 40.00 IU/mL. In the current study, TgAb > 20 IU/mL (functional sensitivity for TgAb assay) was defined as positive, otherwise negative.^[5,36]^

### 2.5 MK measurement

Fasting blood samples were obtained and centrifuged for serum collection, then aliquoted, and stored at –80°C until use. A commercial enzyme-linked immunosorbent assay kit (DuoSet ELISA, R&D Systems Inc.) was used for the measurement of MK concentration (reported as pg/mL). Step 1, 100 μL serum sample or standard was incubated in 96-well microplate (pre-coated with goat anti-human MK antibody) for 2 hours at room temperature. Step 2, after thrice washing, biotinylated goat anti-human MK antibody was added and incubated with captured MK for 2 hours at room temperature. Step 3, after another thrice washing, 100 μL streptavidin-conjugated horseradish-peroxidase were added and allowed to react for 30 minutes in dark place. Step 4, after plate washing, substrate solutions (1:1 mixture of H_2_O_2_ and tetramethylbenzidine) were added to the wells (100 μL in each well) for a 20-minute reaction. Step 5, 1 mol/L H_2_SO_4_ (stop solution) was added (50 μL in each well), and the optical densities of the wells were measured at 450 nm with a Multiskan MS Plate Reader (Labsystems, Helsinki, Finland). Step 6, concentrations of the samples were determined after creating a standard curve by using a 4 parameter logistic curve-fit method.

Though the exact normal level of MK has not been unanimously determined, the normal distribution of serum MK level in healthy people was considered to be between 0 and 625 pg/mL (with a mean value of approximately 253 pg/mL).^[12]^ In our previous work, we found a normal level of MK of 255.01 ± 126.78 pg/mL.^[17]^

### 2.6 Patient group stratification

The PTC patients were stratified according to the degree of dynamic changes of Tg and MK, which method was generally referred to in the TgAb studies ^[27–28]^ with some modifications. The time point for stratification was set at approximately 10 to 12 months after initial ^131^I ablative therapy, during which period another ^131^I therapy was conducted to PTC patients with confirmed ^131^I-avid metastasis, or a diagnostic ^131^I scan was performed to patients without metastasis. For Tg, group 1 was characterized as decreased Tg to an optimal level of less than 1.0ng/mL under thyrotropin stimulation 10 to 12 months after initial ^131^I therapy; group 2 was determined as decreased Tg level, yet not to the optimal level; while group 3 increased Tg level. For MK, group 1 was described as decreased MK to a generally accepted normal level of 255.01 ± 126.78 pg/mL 10 to 12 months after initial ^131^I therapy; group 2 was set as decreased MK, yet not to the normal level; while group 3 MK increased.

### 2.7 Statistics

Data were presented as mean ± standard deviation, median (range), number (percentage) when appropriate. And statistics were analyzed by SPSS 17.0 (SPSS Incorporated, Chicago, IL). Differences of indices between groups of patients were measured by independent samples *t* test or Kruskal–Wallis test when appropriate. Incident difference was assessed by Chi-square test. The Kaplan–Meier method with log-rank test was used to compare metastatic status in disease-free survival curves between groups. Cox regression was adopted to calculate hazard ratio (HR) with 95% confidence interval (CI) in deferent groups. A *P* value not exceeding .05 was considered as statistically significant.

## 3 Results

### 3.1 Patient characteristics at recruitment

During the 4-year period of recruitment, we had a total number of 648 DTC patients who willingly participated in this MK clinical investigation. However, after careful data archive scrutiny, only 241 PTC patients (36 males and 205 females) matched our inclusion and exclusion criteria. The mean age was 47.31 ± 13.06 years (range 17–77 years). The frequency of primary tumor diameter larger than 4 cm, multifocality, extra-thyroidal invasion, and cervical lymph node N1b metastasis (surgery proved lymph node metastases to unilateral, bilateral, or contralateral cervical or retropharyngeal or superior mediastinal lymph nodes, in levels I, II III, IV, V, or VII) from post-surgical histopathology information were 12.86% (31/241 cases), 37.34% (90/241 cases), 42.74% (103/241 cases), and 26.56% (64/241 cases), respectively. The TNM (tumor, lymph node, metastasis—a classification system from The American Thyroid Association ^[3]^) staging at the time of initial ^131^I therapy just after surgery were 91 cases in stage 1 (37.76%), 97 in stage 3 (40.25%), and 53 in stage 4 (21.99%).

### 3.2 Disease status during follow-up

Among the 241 PTC patients, 55 cases had metastatic status (8 males and 47 females) during their respective follow-up period. Age in PTC patients with metastases was significantly higher than patients without. However, there was no obvious sex difference on the metastatic status. All the other parameters showed significant differences between PTC patients with metastases and without metastases (Table 1). The metastatic site distributions of the 55 patients were cervical lymph node in 49 cases (6 males and 43 females) and lung metastases in 20 cases (3 males and 17 females). There were 14 cases with both cervical lymph node and lung metastases (1 male and 13 females).

**Table 1:**
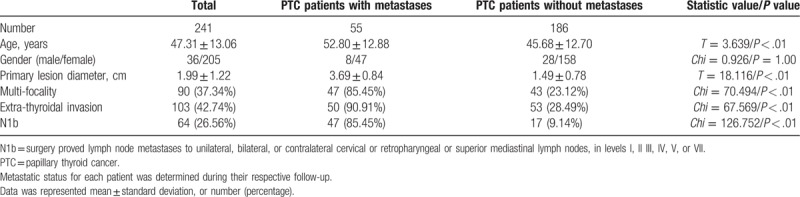
Data comparisons in patients with PTC.

### 3.3 Correlation of disease status with dynamic changes of Tg and MK

The changes in Tg and MK between the time of remnant ablation (Tg1, MK1) and 10 to 12 months thereafter (Tg2, MK2) were evaluated. Based on Tg dynamic change stratification, PTC patients were divided into 3 groups. Age was higher in group 3 than any of the other groups. Characteristics of the primary lesions were significantly worse in group 3. The incidents of metastases during follow-up were significantly higher in group 3 as well (Table 2). Similarly, based on MK dynamic change stratification, PTC patients were divided into 3 groups. The same phenomenon was identified. PTC patients in group 3 were older and had significantly worse primary lesion characteristics than any of the other groups. Higher rates of metastases were also identified in group 3 (Table 3). However, the inter-group differences were higher in Tg than in MK, since the statistic values were generally higher in Table 2 than in Table 3.

**Table 2:**
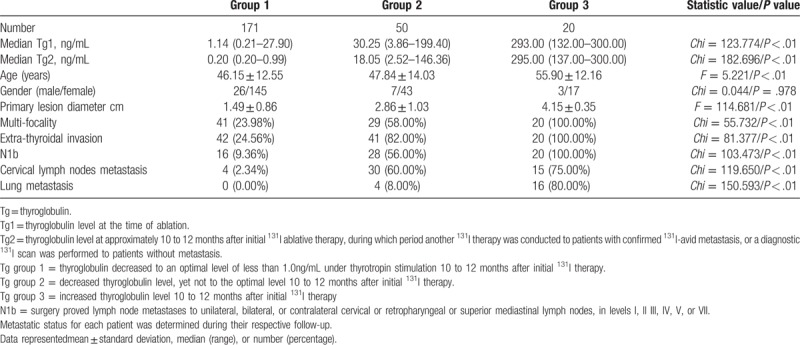
Association of disease status with changes of Tg level between the time of ablation and 10 to 12 months thereafter.

**Table 3:**
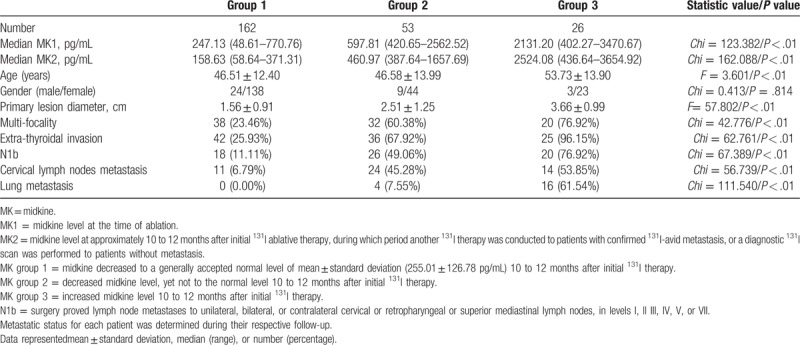
Association of disease status with changes of MK level between the time of ablation and 10 to 12 months thereafter.

### 3.4 Prognostic capabilities of dynamic changes of Tg and MK

**Figure 1:**
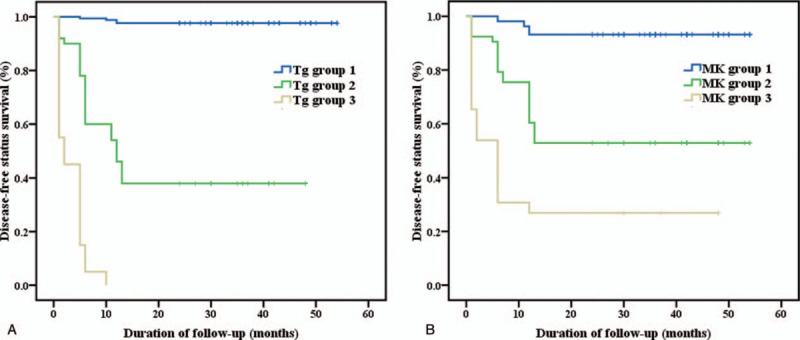
(A) Clinical outcome according to serum thyroglobulin level changes 10 to 12 months after initial ^131^I therapy. (B) Clinical outcome according to serum midkine level changes 10 to 12 months after initial ^131^I therapy. Tg group 1 = thyroglobulin decreased to an optimal level of less than 1.0ng/mL under thyrotropin stimulation 10 to 12 months after initial ^131^I therapy. Tg group 2 = decreased thyroglobulin level, yet not to the optimal level 10 to 12 months after initial ^131^I therapy. Tg group 3 = increased thyroglobulin level 10 to 12 months after initial ^131^I therapy. MK group 1 = midkine decreased to a generally accepted normal level of mean ± standard deviation (255.01 ± 126.78 pg/mL) 10 to 12 months after initial ^131^I therapy. MK group 2 = decreased midkine level, yet not to the normal level 10 to 12 months after initial ^131^I therapy. MK group 3 = increased midkine level 10 to 12 months after initial ^131^I therapy.

Significantly diverse metastatic status in different groups was better illustrated by Kaplan–Meier method in disease-free survival curves (Figure 1). For Tg, significantly higher cumulative rate of disease-free status was found in Tg group 1, yet significantly lower rate was identified in Tg group 3, and the log-rank statistical value was 272.468 (*P* < .01). For MK, the same pattern was displayed with a log-rank statistical value to be 112.740 (*P* < .01). HR was calculated by Cox regression, and compared with Tg group 1, patients in Tg group 2 and 3 possessed high HRs of 12.554 and 19.461 respectively (*P* < .01), while other parameters such as primary tumor diameter and cervical lymph node N1b metastasis also indicated certain prognostic values (Table 4). Similarly for MK, significantly increased HRs of 3.006 and 5.030 were demonstrated in MK group 2 and 3 when compared with MK group 1 (Table 4).

**Table 4:**
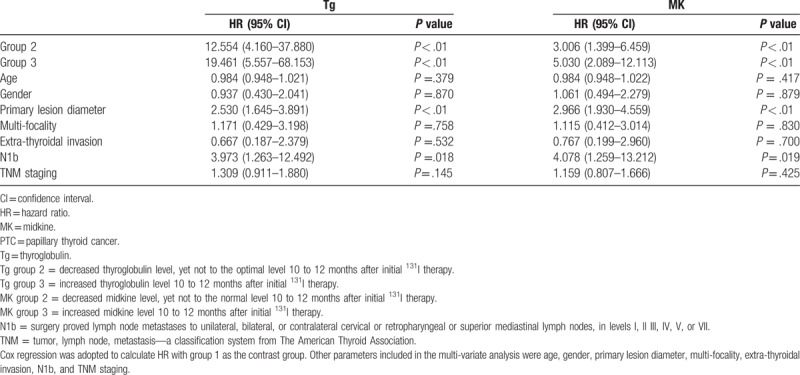
HR of parameters for progostic value of PTC disease status.

## 4 Discussion

MK, a heparin-binding growth factor, was first discovered as a highly expressed factor in embryonic development.^[21]^ Since then, MK has been reported to possess many traits of cancer.^[10–11]^ And opportunities to employ MK as a cancer biomarker exists throughout the disease history of malignancy.^[12]^ In fact, overexpressed MK has been identified in at least 20 different types of cancer.^[12]^ The mechanism by which MK induces tumorigenesis has been proposed to be related to cancer cell proliferation, cell survival, anti-apoptosis, tumorigenesis, and epithelial-mesenchymal transition.^[10–11]^ These functions of MK are mediated by pathways such as the mitogen-activated protein kinase (MAPK), phosphatidylinositol 3-kinase (PI3K)/Akt, and extracellular signal-regulated kinase 1/2 (ERK 1/2), through the MK receptors like anaplastic lymphoma kinase (ALK), and protein tyrosine phosphatase ζ (PTP ζ).^[10–11]^

Because MK is a soluble and secreted protein, serum MK concentration is usually strikingly increased in MK-expressing tumors. Furthermore, a number of studies have demonstrated that MK or a combined biomarker test including MK could outperform other current serum biomarkers in terms of sensitivity for early detection of malignancies, such as hepatocellular carcinoma,^[22–24]^ colorectal carcinoma,^[25]^ esophageal squamous cell carcinoma.^[26]^ For instance, in hepatocellular carcinoma, a comprehensive clinical study from China ^[22]^ published in 2013 enrolled 933 participants, which showed the sensitivity of MK was much higher than that of alpha-fetoprotein (86.9% vs. 51.9%) while their specificities (83.9% and 86.3%) were similar. Notably, serum MK had an outstanding performance in differentiating alpha-fetoprotein negative hepatocellular carcinoma from controls. In 2015, an Egyptian research ^[23]^ confirmatively demonstrated that the sensitivity of MK was significantly higher than that of alpha-fetoprotein in diagnosing hepatocellular carcinoma. Sensitivity of MK was even better in patients with negative alpha-fetoprotein. A very recent Australian investigation ^[24]^ compared serum MK, alpha-fetoprotein, dickkopf-1, and osteopontin for hepatocellular carcinoma diagnosis, which revealed a complementary role of MK and alpha-fetoprotein for hepatocellular carcinoma detection, yet an increased diagnostic power in alpha-fetoprotein negative situation was found for MK only, rather than other biomarkers.

The topic of MK's value in thyroid cancer has been receiving high attentions in recent years. Several immunohistochemical studies ^[13–16]^ on PTC displayed enhanced MK expression in thyroid cancerous tissues, which is the basis for later serological studies or nodular aspiration studies. Kuzu et al ^[18]^ demonstrated that not only serum MK but also nodular MK could predict tumorigenesis of highly suspicious thyroid nodules, serving as an alternative thyroid cancer biomarker. Jee et al ^[19]^ intended to explore the possibility of MK level in the aspirated samples (normalized to Tg concentration) to evaluate thyroid nodules, and found MK/Tg ratio in PTC was greater than in benign thyroid nodules, raising the possibility that this approach might provide adjunctive diagnostic or prognostic values. Our team performed a serum MK study, comparing the diagnostic capabilities of MK and Tg for DTC.^[17]^ A better diagnostic capability of MK than Tg was found to differentiate thyroid nodule malignancy before surgery. However, pre-^131^I-ablative Tg possessed a better metastatic prediction power than MK. DTC patients with MK or Tg levels higher than thresholds (500 pg/mL or 20 ng/mL) would eventually develop a significantly worse ^131^I-avid metastasis-free survival (*P* < .01). The situation of positive TgAb invalidating Tg is somewhat like alpha-fetoprotein negative scenario in hepatocellular carcinoma. Our most recent publication tried to tackle this particular issue.^[20]^ We proved that MK could be a surrogate biomarker when Tg was not suitable for the disease. MK was able to predict DTC metastases with a cut-off MK value of 550.18 pg/mL, a diagnostic accuracy of 83.44%, and an area under the curve value of 0.856 (*P* < .001).

As 1 of the key features of cancer-related circulating MK, when tumor lesions are surgically removed serum MK concentration usually decreases, but will increase its level or remain in high level if recurrence, persistence or metastasis happen.^[11–12]^ For example, Zhu et al ^[22]^ showed that after curative resection, MK level significantly decreased in hepatocellular carcinoma. However, at the time of tumor recurrence MK level was elevated again. Therefore, to our opinion, whether serum MK could be a good surveillance biomarker for PTC is an important clinical matter worthy of investigating, which is the question we tried to answer in the present study. The design of the present research was generally referred to several inspirational studies validating dynamic TgAb changes in DTC,^[27–28]^ in which 3-group TgAb stratification was based on the change of decreased TgAb level to more than 50% about 1 year later, decreased less than 50%, and increased level. In the present study, we modified the grouping methods to fit in our purpose. We found if Tg2 did decrease (compared with Tg1), but not to an optimal level, the risk of metastases was 12.554 times more than if Tg2 could decrease to the optimal level (less than 1.0 ng/mL under thyrotropin stimulation) 10 to 12 months after initial ^131^I therapy. Moreover, if Tg2 increased (compared with Tg1), the risk was 19.461 times higher. As for MK, if MK2 level decreased (compared with MK1), but not to a normal level, the risk of metastases was 3.006 than if it could decrease to a normal level. If MK2 level increased (compared with MK1), it would be 5.030 likely to had metastases. Our results indicated that MK could be regarded as a viable monitor biomarker for DTC, although inferior to Tg. As we indicated previously,^[20]^ MK should theoretically be more suitable in TgAb positive circumstances, which will be 1 of our next stage research projects in the future.

There are 2 major limitations to the study. First, although very sensitive, elevated serum MK has low specificity to any particular oncology type, and a number of other medical conditions might influence MK level as well. We believe this is the most obvious shortcoming for MK. As pointed out by Jones ^[12]^ in his review, 1 strategy to overcome this limitation is to measure MK in conjunction with other known and specific biomarkers. For our research, we studied and compared MK with Tg. In addition, an important way to circumvent MK-influencing co-morbidities is to implement stringent exclusion criteria in order to study relationship between MK and cancer in purpose like the current investigation. This is because MK is involved in various kinds of diseases besides oncology. This rule was strictly abided by in the current presentation. Second, MK is not only a serum biomarker suitable for cancer monitoring; it is also a novel strategical treatment target. Several previous papers showed that inhibiting MK by small interfering RNA could exert obvious therapeutic effects in cancer models.^[29–30]^ At the same time, MK inhibitors were also being explored,^[31]^ which have been successfully applied to several cancer models recently as well.^[32–34]^ Major underlying mechanism involved suppressing MAPK, PI3K, or ERK 1/2 pathways through ALK or PTP ζ receptors. Therefore, it would be very interesting to study the therapeutic efficacy of inhibiting MK in thyroid cancer as a mono-treatment or combined treatment approaches. This aspect of MK was not mentioned in the present study but should be regarded as 1 of the excellent projects in the future.

## 5 Conclusions

We discover that MK can potentially be used as a disease monitoring biomarker for thyroid cancer, although Tg is superior for such a purpose. Further study in TgAb positive circumstance should be conducted to confirm the current findings, and to further MK application. And more importantly, therapeutic strategy targeting MK should be investigated.

## Author contributions

**Conceptualization:** Zhaowei Meng, Ke Xu.

**Data curation:** Ning Li, Chunmei Zhang, Xianghui He, Qiang Jia, Xue Li, Xiangxiang Liu, Xiaoran Wang.

**Formal analysis:** Ning Li, Chunmei Zhang.

**Funding acquisition:** Zhaowei Meng.

**Investigation:** Ning Li, Chunmei Zhang, Zhaowei Meng, Xianghui He, Yang Yu, Qiang Jia, Xue Li, Xiangxiang Liu, Xiaoran Wang.

**Methodology:** Zhaowei Meng, Ke Xu.

**Project administration:** Zhaowei Meng, Ke Xu.

**Writing – original draft:** Ning Li, Chunmei Zhang, Qiang Jia, Xue Li, Xiangxiang Liu, Xiaoran Wang.

**Writing – review & editing:** Zhaowei Meng, Ke Xu, Xianghui He, Yang Yu.
